# Climates of the past

**Published:** 1895-11-30

**Authors:** 


### MODERN SOCIOLOGY.

### CLIMATES OF THE PAST.

THAT the climate of the earth was once very different from what it is now is a thing which every schoolboy, not necessarily a budding Macaulay, knows. But wiser men than he cannot do more than guess at the causes by which such climatic variations come about. Some geologists attribute them to temporary displacements of the poles, and, indeed, there is an impression abroad of a coquettish earth, whirling about on a variable axis, and mixing up the equator and the poles in hopeless confusion. Certain it is—for there are some inferences so obvious that we may take them as certainties—that within the arctic circle are to be found fossil flora akin to those of existing South American forests, while in the torrid zone of to-day are evidences of glacial action; and the remains of the great reptiles which are discovered show that at one time there was on the earth a great heat—greater probably than any we have now, for reptiles have less heat-generating power in themselves than other creatures, such as the birds and mammals, and depend more than these on the warmth of the sun.

These facts being admitted, what was the cause of them? An ingenious book, by M. Eugene Dubois, who dates his preface from Toeloeng-Agoeng, in Java, entitled "The Climates of the Geological Past," flings overboard all explanations based on the hypothesis of the variable pole. M. Dubois points out in the first place that the mere power of acclimatisation will account for some of the phenomena which geologists have observed. Both plants and animals not merely exist, but often flourish, in climates to which they are not indigenous. Put a few rabbits in Australia, and presently they will so multiply that a price will be put on their heads. Put a palm in a Russian hothouse, and if it has warmth enough it will put up with the short winter day of the north, and not pine for the brilliant equatorial glare. Therefore it does not follow that climatic conditions were as wholly different from the present as some have supposed because the swamp cypress, which now lives in the Southern States of America, is found in a fossil state in 81¾ deg. north latitude, and similar surprises meet us in every geologic age. Mere acclimatisation will not indeed account for such phenomena, but neither is it necessary to assume that the poles were once the equator and the equator the poles.

M. Dubois explains it all by the evolution of the sun. The sun is at present a yellow star. Astronomers tell us that yellow stars are in their second stage of evolution from the nebula whence they originate to their final extinction. The sun's energy is derived from the gradual shrinking of its gaseous body, and its first stage, when its condition was more gaseous and its heat more intense, is already past. At that period it would burn with a white or rather bluish-white light, such as now characterises the star Sirius; it would possess a hot, dense atmosphere of hydrogen, and its yearly loss of heat must have been much greater in the white stage than now, and our earth, therefore, much warmer.

This will explain the hotter periods of our earth's history, but how will it affect the colder ones? M. Dubois explains that variations of climate are produced at the present day by winds and currents. Our own islands profit by the Gulf Stream, and, on the other hand, the warm coasts of South America are rendered more tolerable by the cold current of Peru. We can conceive of the bleak wastes of Siberia becoming fertile plains if a range of mountains intervened to protect them from the bleak winds of the Arctic Ocean. The fact that at the present moment places on the same parallel of latitude have such different climates makes it not impossible that similar conditions prevailed in the past, and the remains of old glaciers by no means prove an Arctic climate in the places where they are found. Cold is a factor, but not the only factor, in the production of glaciers. In a moist climate we may find glaciers with a mean temperature, which in a drier air would be impossible. In New Zealand to-day this is seen where, with a mean yearly temperature equal to the climate of Vienna, but very damp, glaciers descend to within a few hundred yards of the sea, making their slow chill way between forests thick with tree-ferns, palms, and fuchsias; and in the more familiar Alps it has been observed that glaciers may increase in warm years if only the prevailing winds are moist west ones from the Atlantic. M. Dubois, therefore, infers that in the Pleistocene period, when the intenser heat of the sun caused a greater evaporation from the earth, especially within the tropics, comparatively slight diminutions of land temperature caused a considerable increase of old glaciers and originated new ones.

When the sun was passing from the first to the second stage of its star-life, from the white to the yellow heat, there must have been a comparatively quick reduction of the earth's temperature which would produce great geological changes. In the warmer ages the wind currents laden with equatorial heat made their way polewards, modifying the otherwise natural climate in the same way as now and to a greater extent. But when in the cooling of the sun the tropics could no longer spare so much of their heat, the temperate and arctic zones, getting less of either direct or borrowed heat, had their climates suddenly changed, so that old organisms, both animal and vegetable, that were unable to adapt themselves to the changed conditions disappeared, and new ones, more suited to their surroundings, were evolved.

Our earth's existence as "the habitable globe" therefore depends, in M. Dubois' opinion, on the gradual shrinkage of the sun. This is a depressing thought, for when the sun goes, where shall we be? But we need have no immediate anxiety. Three-fifths of the sun's life is already past, but it no longer spends it heat so lavishly as in its hot youth, and physicists calculate that it will exist as a luminous and heat-giving body for five or six million years to come. For that time then it may be the home of us and our descendants. After that posterity must look after itself.

